# Integrative Analyses Followed by Functional Characterization Reveal *TMEM180* as a Schizophrenia Risk Gene

**DOI:** 10.1093/schbul/sbab032

**Published:** 2021-03-26

**Authors:** Jun-Yang Wang, Xiao-Yan Li, Hui-Juan Li, Jie-Wei Liu, Yong-Gang Yao, Ming Li, Xiao Xiao, Xiong-Jian Luo

**Affiliations:** 1 Key Laboratory of Animal Models and Human Disease Mechanisms of the Chinese Academy of Sciences & Yunnan Province, Kunming Institute of Zoology, Chinese Academy of Sciences, Kunming, Yunnan 650223, China; 2 Kunming College of Life Science, University of Chinese Academy of Sciences, Kunming, Yunnan 650204, China; 3 KIZ-CUHK Joint Laboratory of Bioresources and Molecular Research in Common Diseases, Kunming Institute of Zoology, Chinese Academy of Sciences, Kunming, Yunnan 650223, China; 4 Center for Excellence in Animal Evolution and Genetics, Chinese Academy of Sciences, Kunming, Yunnan 650223, China; 5 CAS Center for Excellence in Brain Science, Chinese Academy of Sciences, Shanghai 200031, China

**Keywords:** schizophrenia, *TMEM180*, integrative analysis gene expression, TWAS, eQTL

## Abstract

Recent large-scale integrative analyses (including Transcriptome-Wide Association Study [TWAS] and Summary-data-based Mendelian Randomization [SMR]) have identified multiple genes whose *cis*-regulated expression changes may confer risk of schizophrenia. Nevertheless, expression quantitative trait loci (eQTL) data and genome-wide associations used for integrative analyses were mainly from populations of European ancestry, resulting in potential missing of pivotal biological insights in other continental populations due to population heterogeneity. Here we conducted TWAS and SMR integrative analyses using blood eQTL (from 162 subjects) and GWAS data (22 778 cases and 35 362 controls) of schizophrenia in East Asian (EAS) populations. Both TWAS (*P* = 2.89 × 10^–14^) and SMR (*P* = 6.04 × 10^–5^) analyses showed that decreased *TMEM180* mRNA expression was significantly associated with risk of schizophrenia. We further found that *TMEM180* was significantly down-regulated in the peripheral blood of schizophrenia cases compared with controls (*P* = 8.63 × 10^–4^ in EAS sample), and its expression was also significantly lower in the brain tissues of schizophrenia cases compared with controls (*P* = 1.87 × 10^–5^ in European sample from PsychENCODE). Functional explorations suggested that *Tmem180* knockdown affected neurodevelopment, ie, proliferation and differentiation of neural stem cells. RNA sequencing showed that pathways regulated by *Tmem180* were significantly enriched in brain development and synaptic transmission. In conclusion, our study provides convergent lines of evidence for the involvement of *TMEM180* in schizophrenia, and highlights the potential and importance of resource integration and sharing at this big data era in bio-medical research.

## Introduction

Schizophrenia is a severe mental disorder imposing great economic and societal burden.^1^ High heritability indicates a dominant role of genetic risk factors in schizophrenia,^2^ and over 200 risk loci have been reported by genome-wide association studies (GWASs).^3–12^ Despite that GWASs have made unprecedented achievements in the past decade, deciphering the genetic underpinnings and pathophysiology of schizophrenia is still challenging owing to the genetic heterogeneity of the disease between continental populations^13–15^ and the unclear functionality of most GWAS loci.

Recent endeavors to overcome these challenges have achieved prominent success. For example, GWASs performed in populations of East Asian (EAS),^8,9,11^ Indian,^16^ African and Latino ancestries^17^ have identified novel risk loci for schizophrenia. In addition, integrative studies using European data have identified potential target genes of multiple schizophrenia risk variants.^18–27^ Although these studies have provided novel biological insights, almost all of these integrative analyses utilized genome-wide associations and eQTL data of populations of European ancestry, potentially missing pivotal genetic and biological insights in other populations. To overcome the deficiency of integrative analyses in non-European populations and to illuminate the potential roles of the identified risk genes in schizophrenia, in this study, we firstly conducted large-scale integrative analyses (ie, TWAS and Summary-Data-Based Mendelian Randomization [SMR]) using reported genome-wide associations and eQTL data of population of East Asian ancestry (EAS). We then explored if risk genes identified by integrative analyses were dysregulated in schizophrenia cases compared with controls. We also investigated the role of the identified risk gene (ie, *Tmem180*) in neurodevelopment by using neural stem cell model. Finally, we investigated the genes and pathways potentially regulated by *Tmem180* using transcriptome sequencing. Our study suggests that *TMEM180* is a schizophrenia risk gene whose expression alternation may have a role in schizophrenia (through affecting neurodevelopment and schizophrenia-associated biological pathways).

## Materials and Methods

### Genome-Wide Associations of EAS

Genome-wide SNP associations in EAS were retrieved from a recent schizophrenia GWAS.^11^ Briefly, Lam et al conducted the largest schizophrenia GWAS (22 778 cases and 35 362 controls) in EAS and identified 21 genome-wide significant associations at 19 loci.^11^ Detailed information about the EAS GWAS can be found in the original paper.^11^

### eQTL Data of EAS

Recent studies have revealed differences in genetic architecture of gene expression in different populations,^28,29^ indicating the importance of conducting integrative analyses using genetic associations and eQTL data from the same population (ie, if GWAS associations were from EAS, it is better to use eQTL data from EAS). We used eQTL data from lymphoblastoid cell lines of EAS populations (162 donors) in this study.^30^ Detailed information about eQTL data of EAS are provided in the supplementary methods.

### Transcriptome-Wide Association Study

To identify genes whose cis-regulated expression changes are associated with risk of schizophrenia, we performed a Transcriptome-Wide Association Study (TWAS) by integrating GWAS associations and eQTL data. The TWAS analysis was performed using the FUSION software^31^ (http://gusevlab.org/projects/fusion/). Detailed information about TWAS are provided in the supplementary methods.

### SMR Analysis

We used *SMR* integrative analysis approach developed by Zhu *et al*. to identify schizophrenia risk genes through integrating eQTL data and GWAS signals.^32^ Details about the SMR analyses can be found in the original paper^32^ and are provided in the supplementary methods.

### Functional Annotation of rs2902544

We explored the functionality of rs2902544 using functional annotation tools RegulomeDB^33^ and Alibaba2^34^. Detailed information about functional annotation are provided in the supplementary methods.

### Expression Analysis of *TMEM180* in Peripheral Blood of Schizophrenia Cases and Controls (EAS Sample)

TWAS identifies disease-associated genes under the assumption that genetic variations confer risk of disease by modulating gene expression.^31^ To further explore if the schizophrenia risk gene *TMEM180* identified by TWAS and SMR integrative analyses in EAS was dysregulated in schizophrenia cases, we examined gene expression level of *TMEM180* in peripheral blood of schizophrenia cases and controls by using the expression data from the study of Sun et al.^35^ More detail information about schizophrenia diagnosis, blood collection, RNA extraction, quality control, and statistical analysis were provided in supplementary material and can be found in the original publication.^35^

### Expression Analysis of *TMEM180* in Brain Tissues of Schizophrenia Cases and Controls (European Sample)

We further examined *TMEM180* mRNA expression level in brains of schizophrenia cases and controls. As there is no publicly available Asian brain expression data for analysis, we used European brain expression data from the PsychENCODE^21^ for *TMEM180* expression analysis. We extracted the expression values (fragments per kilobase of transcript per million mapped reads (FPKM)) and *P* value of *TMEM180* from PsychENCODE website. Detailed information about the study subjects are provided in supplementary material and can be found in the related publication.^21^

### Isolation and Culture of Mouse Neural Stem Cells (mNSCs)

We isolated mNSCs according to the published protocols^36,37^ with some minor modifications as described in our recent study.^38^ In brief, brains of mouse embryos (embryonic day 13.5 (E13.5), C57BL/6) were dissected under microscope to obtain neural stem cells from the ventricular zone (VZ) and sub-ventricular zone (SVZ) tissues. Details about isolation and culturing of mNSCs are provided in supplementary material.

### Knockdown Experiments

The short hairpin RNAs (shRNAs) targeting mouse *Tmem180* were designed using BLOCK-iT™ RNAi Designer (https://rnaidesigner.thermofisher.com/rnaiexpress/sort.do) (supplementary table 1). Detailed procedures were provided in supplementary methods.

### Proliferation Assays of mNSCs

Proliferation assays (including EdU incorporation and CCK-8) were performed as previously described^38^ and detailed procedures were provided in supplementary methods.

### Differentiation of mNSCs Into Neurons and Astrocyte Cells

The mNSCs cells were seeded onto the 24-well plates at a density of 2 × 10^5^ cells/well (pre-coated with laminin [SIGMA, Cat.No: L2020-1mg]) and cultured in proliferation medium. After one day, the proliferation medium was replaced with differentiation medium. Differentiation assays were performed as previously described^38^ and detailed procedures were provided in supplementary methods.

### Immunofluorescence Staining

Detailed procedures about immunofluorescence staining are provided in supplementary material. The primary and secondary antibodies used in this study were provided in supplementary material.

### Real-Time Quantitative PCR

RNA was extracted with TRIzol RNA Isolation Reagents (Life technologies, 15596018) according to the manufacturer’s instructions. Detailed information about procedures and analyses of qPCR are provided in the supplementary methods. Primers sequences are listed in supplementary table 1.

### Transcriptome Analysis

Detailed procedures about transcriptome analysis (RNA sequencing) are provided in supplementary material.

## Results

### TWAS and SMR Integrative Analyses in EAS Identified *TMEM180* as a Schizophrenia Risk Gene

**Fig. 1.**
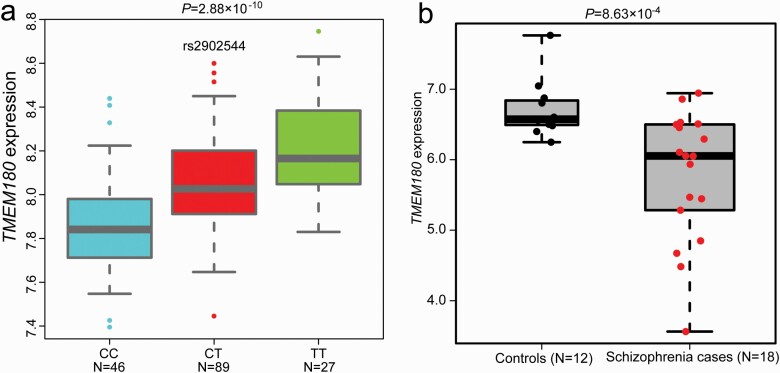
Expression quantitative trait loci and *TMEM180* expression analyses. (a) The schizophrenia risk allele of rs1902544 is associated with lower *TMEM180* expression in EAS (effect size (beta) = 0.182). (b) *TMEM180* expression was significantly down-regulated in schizophrenia cases compared with controls (with the effect size [Cohen’s *d*] of 1.22).

To prioritize candidate genes whose expression alterations may confer risk of schizophrenia, several integrative analyses have been performed.^18,22,23,25,27,31,32,39,40^ However, most of the integrative analyses were conducted in populations of European ancestry. In this study, we performed integrative analyses using genome-wide associations of schizophrenia (22 778 schizophrenia cases and 35 362 controls) and eQTL data (162 individuals) from populations of EAS ancestry.^11,30^ We first conducted a TWAS31 in EAS and identified 4 transcriptome-wide significant risk genes (including *TMEM180*, *ACTR1A*, *SFXN2,* and *MAD1L1*) for schizophrenia (corrected by Bonferroni multiple comparison testing) (table 1), and *TMEM180* showed the most significant association (TWAS *P* = 2.89 × 10^–14^). SNP rs2902544 showed significant association with schizophrenia and *TMEM180* expression (figure 1a). Of note, functional annotation suggested that rs2902544 may be a functional variant (supplementary figure 1). We further performed another integrative analysis (ie, SMR32) by using the same GWAS and eQTL data as the TWAS analysis. SMR integrative analysis identified 2 schizophrenia risk genes (*SFXN2* and *TMEM180*) (corrected by Bonferroni multiple comparison testing) (table 2). Nevertheless, HEIDI (heterogeneity in dependent instruments) test^32^ showed that *SFXN2* could not pass heterogeneity test (*P*_HEIDI_ < 0.05), suggesting that the association between *SFXN2* and schizophrenia might due to linkage or pleiotropic effect (rather than causal effect). Thus, the only significant risk gene identified by SMR is *TMEM180* (*P* = 6.04 × 10^–5^). Collectively, both TWAS and SMR integrative analyses supported that *TMEM180* was significantly associated with schizophrenia.

**Table 1.** Transcriptome-Wide Significant Schizophrenia Risk Genes Identified by TWAS in EAS

Gene	CHR	Best.GWAS.ID^a^	A1	A2	OR^b^	eQTL ID^c^	TWAS.Z^d^	TWAS.P
***TMEM180***	**10**	**rs4147157**	**A**	**G**	**0.89**	**rs2902544**	**−7.603**	**2.89E-14**
*ACTR1A*	10	rs4147157	A	G	0.89	rs284860	−5.2973	1.18E-07
*SFXN2*	10	rs4147157	A	G	0.89	rs2902548	5.0379	4.71E-07
*MAD1L1*	7	rs10239050	A	G	1.07	rs1107592	4.647	3.37E-06

*Note:*^a^The SNP that showed the most significant association with schizophrenia in this locus.

^b^Odds ratio is based on A1.

^c^The SNP that showed the most significant association with gene expression in this locus.

^d^The *Z* statistic reflects the association strength between this gene and schizophrenia. *Z*<0 suggests that this gene was predicted to be down-regulated in schizophrenia cases compared with controls, and vice versa. Transcriptome-wide significant (Bonferroni corrected P <0.05) gene is shown in bold.

**Table 2.** Schizophrenia Risk Genes Identified by SMR Integrative Analysis in EAS

Gene	Chr	Top SNP	Top SNP_Chr	A1	A2	OR^a^	HEIDI_P^b^	SMR_P
*SFXN2*	10	rs2902548	10	T	C	0.92	2.27E-03	2.52E-06
***TMEM180***	**10**	**rs17114641**	**10**	**T**	**G**	**1.10**	**8.22E-02**	**6.04E-05**

*Note:*^a^Odds ratio is based on A1.

^b^HEIDI (heterogeneity in dependent instruments) test was used to distinguish pleiotropy from linkage. If a gene passes HEIDI test (*P*>0.05), suggesting that there is a single causal variant influencing both disease risk and gene expression. Thus, the expression change of this gene may have a role in disease susceptibility. Transcriptome-wide significant (Bonferroni corrected P <0.05) gene is shown in bold.

### Risk Allele of rs2902544 was Associated With Lower *TMEM180* Expression

Our TWAS analysis showed that rs2902544 was simultaneously associated with schizophrenia (*P* = 3.45 × 10^–13^) and *TMEM180* expression (*P* = 2.88 × 10^−10^) in EAS (table 1), suggesting that genetic variation may confer schizophrenia risk by regulating *TMEM180* mRNA expression. Further analysis showed that the risk allele (ie, C allele) of rs2902544 was associated with lower *TMEM180* expression (figure 1a), implying that risk variants might contribute to schizophrenia risk through down-regulating *TMEM180*.

### Down-Regulation of *TMEM180* in Schizophrenia Cases Compared With Controls

As stated above, TWAS and eQTL analyses of rs2902544 predicted down-regulation of *TMEM180* in schizophrenia cases compared with controls (table 1). We then examined *TMEM180* mRNA expression changes between schizophrenia cases and controls using the expression data from Sun *et al*. (Chinese sample).^35^ Consistent with the prediction of integrative analyses, we found that *TMEM180* was significantly down-regulated in the blood samples of schizophrenia cases compared with controls (*P* = 8.63 × 10^–4^) (figure 1b), with an effect size (Cohen’s *d*) of 1.22.

We further explored *TMEM180* mRNA expression in brains of schizophrenia cases and controls using expression data from the PsychENCODE.^41^ Again, *TMEM180* was significantly down-regulated in the brains of schizophrenia cases compared with controls (*P* = 1.87 × 10^–5^), with an effect size (Cohen’s *d*) of 0.906. These consistent results from different samples and tissues suggested that dysregulation of *TMEM180* might play a role in schizophrenia.

### Knockdown of *Tmem180* Affected Proliferation of Mouse Neural Stem Cells

**Fig. 2.**
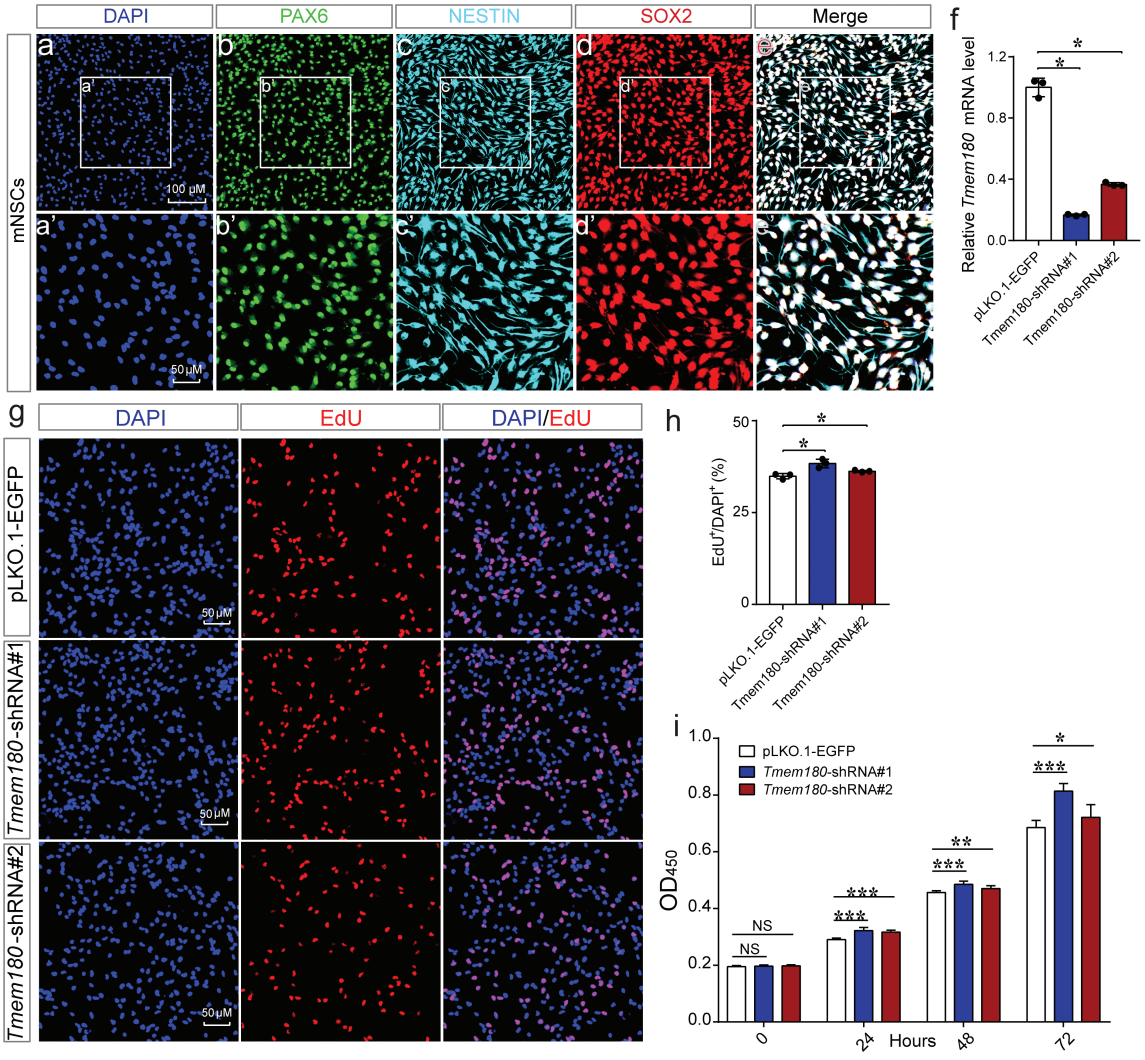
*Tmem180* knockdown promotes proliferation of mNSCs significantly. (a–e) Immunofluorescence staining showed that the isolated mNSCs express 3 well-characterized markers for NSCs, including SOX2, PAX6, and NESTIN, indicating that the cells were NSCs. (f) Expression of *Tmem180* in mNSCs was significantly knocked-down by the designed shRNAs. (g) EdU incorporation assay showed that EdU^+^ (red) cells were significantly increased in *Tmem180* knocked-down cells compared with controls. DAPI^+^ was used to stain the nucleus (blue). (h) The quantification results of the EdU incorporation assay. (i) CCK-8 assay revealed that the *Tmem180* knockdown significantly promote proliferation of NSCs. Data showed at 3 time points, 24, 48 and 72 hours. Two-tailed *Student’s t test* was used to compare if the difference was significant. n = 3 for **f**, *n* = 3 (EdU positive cells were counted from 6 independent immunostaining images for each sample) for **g**, *n* = 9 for **i**. Data are represented as mean ± SD. *P < .05; **P < .01; ***P < .001.

Although the pathophysiology of schizophrenia remains largely unknown, multiple lines of evidence (including genetic^42^ and functional studies^27,43–45^) support the neurodevelopmental hypothesis, which posits that schizophrenia is mainly attributed to abnormal brain development.^46–50^ To mimic the effect of *TMEM180* down-regulation on neurodevelopment, we used the mouse neural stem model, which was frequently used in studying the role of schizophrenia risk genes in neurodevelopment.^27,43–45^ We validated the identity of isolated mNSCs using well-characterized markers, including PAX6, NESTIN and SOX2 (figures 2a–e). We designed 2 shRNAs to knockdown *Tmem180* expression in mNSCs and RT-qPCR showed that *Tmem180* was significantly down-regulated by the shRNAs (figure 2f). Both EdU and CCK-8 assays showed that *Tmem180* knockdown promoted proliferation of mNSCs significantly (figures 2g–i), indicating that *Tmem180* has a role in regulating proliferation of NSCs.

### Knockdown of *Tmem180* Affected Differentiation of mNSCs Into Neuronal and Astrocyte Cells

**Fig. 3.**
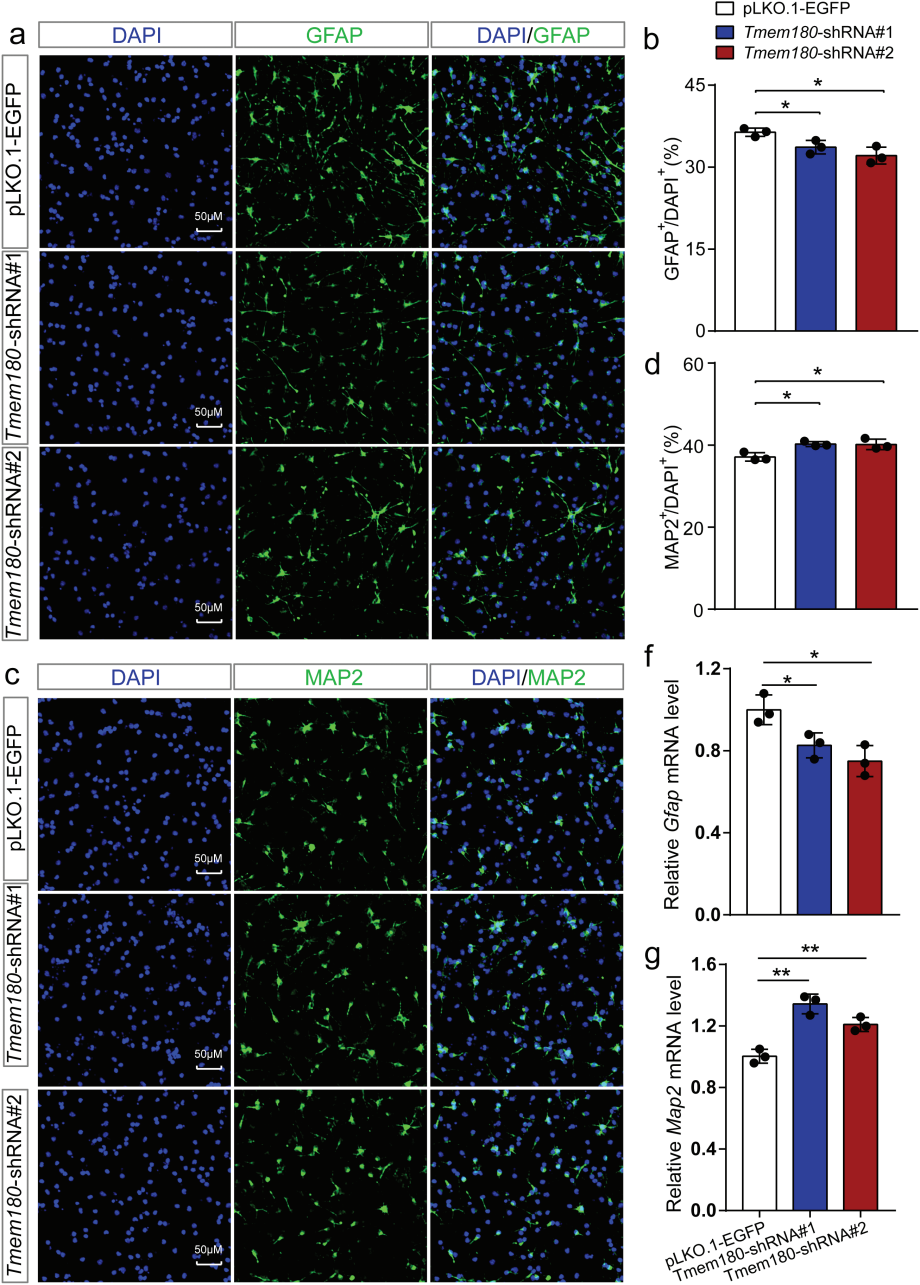
*Tmem180* knockdown affects differentiation of mNSCs. (a) Representative immunofluorescence staining images for GFAP^+^ astrocyte cells (green) and DAPI^+^ (blue). (b) Quantification for the ratio of GFAP positive astrocyte cells in *Tmem180* knockdown and controls mNSCs. The ratio of GFAP positive astrocyte cells was significantly decreased in *Tmem180* knockdown group compared to control group, indicating that the differentiation of mNSCs into astrocyte cells were impaired. (c) Representative immunofluorescence staining images for MAP2^+^ neurons (green) and DAPI^+^ (blue). (d) Quantification for the ratio of MAP2 positive neurons in *Tmem180* knockdown and controls NSCs. The ratio of MAP2 positive astrocyte cells was significantly increased in *Tmem180* knockdown group compared to control group, indicating that the differentiation of NSCs into neurons were enhanced. (f,g) RT-qPCR results showed that *Tmem180* knockdown significantly affected the relative expression level of *GFAP* and *MAP2*. pLKO.1-EGFP was used as controls (ie, these cells were transfected with random shRNAs and EGFP). Two-tailed *Student’s t test* was used to compare if the difference was significant. *n* = 3 (GFAP positive cells were counted from 8 independent immunostaining images for each sample) for **a,***n* = 3 (MAP2 positive cells were counted from 6 independent immunostaining images for each sample) for **c.** *P < .05; **P < .01.

In the early stage of neurodevelopment, the NSCs first undergo serial proliferation and self-renewal in the ventricular zone (VZ) and sub-ventricular zone (SVZ) to generate numbers of NSCs and neural progenitor cells.^51^ With the progress of development, these NSCs and neural progenitors migrate outside and differentiate into different types of neural cells and astrocyte cells. To further explore the role of *TMEM180* in neurodevelopment, we next investigated the role of *TMEM180* in neural differentiation. Compared with control NSCs, we found that the proportion of GFAP positive astrocytes cells (GFAP^+^) was significantly decreased in *Tmem180* knockdown group (figures 3a and 3b). By contrast, the proportion of MAP2 positive neuronal cells (MAP2^+^) was significantly increased (figures 3c and 3d). We validated the impact of *Tmem180* knockdown on neural differentiation with RT-qPCR. Consistent with the immunostaining results, RT-qPCR showed that *Tmem180* knockdown significantly altered the expression of GFAP and MAP2, with the same effect direction as observed in immunostaining assays (figures 3f and 3g). Collectively, these results demonstrate the important role of *TMEM180* in regulating neural differentiation.

### *TMEM180* Regulated Schizophrenia-Associated Pathways

**Fig. 4.**
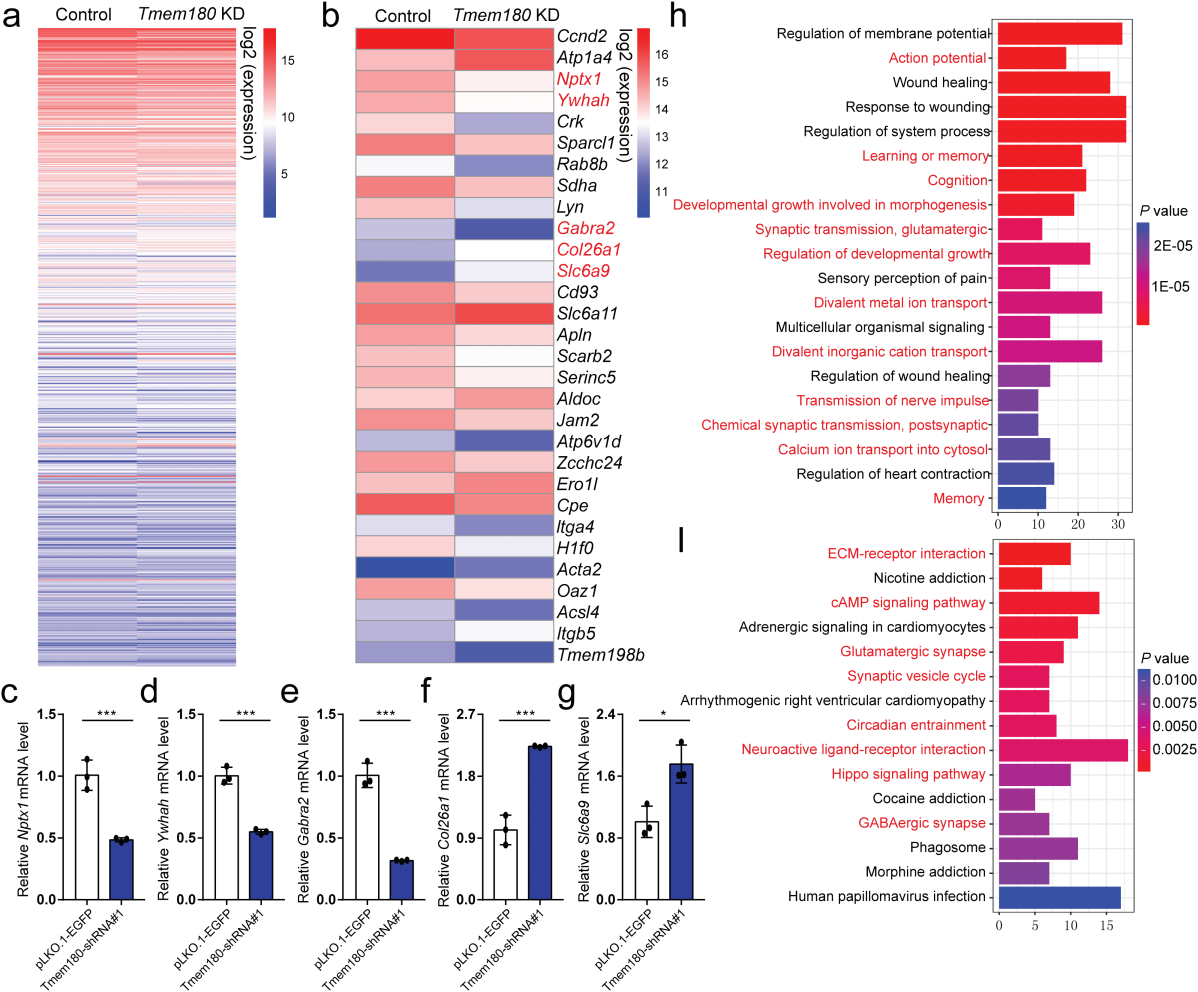
*Tmem180* regulates schizophrenia-associated biological processes and pathways. (a) Expression heatmap of all differentially expressed genes (*n* = 654) identified in *Tmem180* knockdown NSCs compared with controls. (b) Heatmap plot of the top 30 differentially expressed genes. (c–g) qPCR validation of RNA-Seq results. Five genes (marked by red color in **b**) were selected for qPCR verification. All of the 5 genes that showed differential expression by RNA-Seq were validated by RT-qPCR, indicating the reliability of RNA-Seq. (h,i) GO and KEGG analyses of the differentially expressed genes. Pathways marked with red color were previously reported to be associated with schizophrenia. *P* values were calculated by Two-tailed *Student’s t test* was used for statistical test. *n* = 3 for **c–g,** *P < .05; **P < .01.

To further investigate the biological and signaling pathways regulated by *TMEM180*, we performed transcriptome analysis. We conducted RNA-Seq to examine the impact of *Tmem180* knockdown on global gene expression profiling in mNSCs. We identified 654 genes (supplementary table 2) that were differentially expressed (fold change > 1.5 and adjusted *P* < .05) in *Tmem180* knockdown mNSCs (compared with controls) (figure 4a). We selected 5 genes (including *Nptx1*, *Ywhah*, *Gabra2, Col26a1,* and *Slc6a9*) (figure 4b) to validate the results of RNA-seq using RT-qPCR (figures 4c–g), and the selection criteria of these 5 genes were as follows: First, these 5 genes were from the top 30 differentially expressed genes (based on RNA-seq). Second, these genes are abundantly expressed (https://www.proteinatlas.org/)^52^ (supplementary figure 2) and have pivotal roles in the human brain.^52–65^ Detailed information about the roles of these genes in the central nervous system was provided in the supplementary methods. Taken together, these lines of evidence indicated the important role of the potential target genes of *TMEM180* in brain development and psychiatric disorders, suggesting that *TMEM180* may confer risk of schizophrenia through regulating these genes.

We next performed GO analysis to explore if the 654 differentially expressed genes were enriched in specific biological categories or signaling pathways. Our GO analysis showed that the differentially expressed genes were mainly enriched in biological processes associated with schizophrenia, including action potential,^66^ learning or memory,^67,68^ cognition,^69–71^ synaptic transmission, etc (figure 4h). In addition, KEGG pathway analysis showed that the dysregulated genes were significantly enriched in schizophrenia-associated signaling pathways, including ECM-receptor interaction,^72^ cAMP signaling pathway,^73^ glutamatergic synapse, synaptic vesicle cycle,^74–77^ GABAergic synapse,^78,79^ etc (figure 4i). Collectively, our transcriptome analysis showed that *TMEM180* may contribute to schizophrenia by regulating these biological processes and signaling pathways.

## Discussion

In this study, we identified *TMEM180* as a schizophrenia risk gene through integrating genome-wide associations and eQTL data from EAS. We provided convergent lines of evidence that support dysregulation of *TMEM180* might have a role in schizophrenia. First, our TWAS and SMR integrative analyses suggested that *TMEM180* is schizophrenia risk gene whose down-regulation may have a role in schizophrenia. Of note, previous TWAS studies^21,60^ using GWAS associations and brain eQTL data of Europeans did not identify *TMEM180* as a schizophrenia risk gene (supplementary table 3), indicating the necessity and importance of performing integrative analysis using GWAS and eQTL data from non-European populations. Second, consistent with the prediction of integrative analyses, mRNA expression analysis showed that *TMEM180* was significantly down-regulated in peripheral blood of schizophrenia cases compared with controls in EAS sample. Third, *TMEM180* also showed a significant down-regulation in brains of schizophrenia cases compared with controls in European sample from the PsychENCODE,^22^ further supporting the potential involvement of *TMEM180* in schizophrenia. Fourth, we found that *Tmem180* knockdown affected proliferation and differentiation of NSCs, indicating that *Tmem180* is required for normal proliferation and differentiation of NSCs. These results also suggested that *TMEM180* may contribute to susceptibility of schizophrenia by affecting neurodevelopment. Finally, transcriptome analysis demonstrated that *Tmem180* regulates schizophrenia-associated pathways, including pathways related to synaptic transmission, memory and cognition.

*TMEM180* is also known as *MFSD13A* (Major Facilitator Superfamily Domain Containing13A) and it encodes a transmembrane protein which contains 12 transmembrane domains.^80^ Previous studies have showed that *TMEM180* knockdown (with siRNAs) promotes proliferation of the human pancreatic cancer cells.^81^ In addition, *TMEM180* is highly expressed in colorectal cancer cells^80^ and it may be a new marker for colorectal cancer.^82,83^ To date, the exact function of *TMEM180* is still unclear and we know little about the role of *TMEM180* in brain and schizophrenia pathogenesis. Our transcriptome sequencing showed that synaptic transmission and neuronal related pathways were significantly affected by *Tmem180* knockdown, suggesting that *TMEM180* may have a pivotal role in the brain. The potential roles of *TMEM180* in the brain are discussed in the supplementary discussion and related data are provided in supplementary figures 3–5.

Recent integrative analyses have linked schizophrenia risk variants to genes,^18,21,23,25,27,32,60^ thus providing a starting point for further functional characterization and mechanism dissection. These integrative analyses not only translated the genetic associations into risk genes,^24^ but also provided potential insights into schizophrenia pathogenesis. As the genome-wide associations and eQTL data used for integrative analyses were primarily from populations of Europeans, there is a necessity to look at the other continental populations in consideration of the population genetic heterogeneity. Fortunately, recent studies have begun to dissect the genetic architecture of schizophrenia in other populations, including populations of EAS,^9,11^ African and Latino ancestries.^17^ These studies provided important biological insights into the genetic etiology of schizophrenia and are well complementary to the GWASs conducted in European populations. In this study, we reported the first integrative analysis on schizophrenia using genome-wide associations and eQTL data of EAS. Our study identifies *TMEM180* as a novel risk gene for schizophrenia and provides a complementary scheme to the integrative studies performed in European populations. Of note, the original study by Lam *et al*. suggested that *ACTR1A* might be the responsible gene at this locus as *ACTR1A* is the gene nearest the top association (the lead or index) variant at this locus.^11^ Our study highlights that the gene nearest the top association cannot be simply presumed to harbor the causal variations. The risk or causal variants may confer schizophrenia risk through regulating expression of distal genes (rather than the nearest gene). Interestingly, we noticed that *TMEM180* did not show significant association with schizophrenia in previous GWAS10 (supplementary figure 6a) and integrative studies of schizophrenia (supplementary tables 3 and 4) (using European),^21,22,25^ suggesting the potential population specificity of this risk gene. Finally, the frequency of the risk allele (C) of rs2902544 also showed differences in Europeans and East Asians (supplementary figure 7), implying differential power to detect this association across ancestries, and either random drift or possibly positive selection favoring the minor allele in out-of-Africa populations.

Our study also suggests ancestry-specific findings diverge and converge across modalities in schizophrenia. Detailed discussions on this are provided in the supplementary discussion.

There are several limitations of this study. First, the sample size of schizophrenia GWAS included in this study was still relatively small compared to integrative studies performed in European,^22,25^ which may limit the identification of more promising candidate risk genes for schizophrenia. Second, as no brain eQTL data was available for EAS, we used eQTL data from the lymphoblastoid cell lines (as a surrogate) for integrative analysis. Considering that schizophrenia is a mental disorder that is mainly originated from abnormal brain development and function, it is ideal to use eQTL data from brain tissues to conduct integrative analysis. Using eQTL data from non-brain tissues for integrative analyses may miss important information. In fact, only a significant gene (ie, *TMEM180*) was identified in our study. The relatively small sample size included in EAS GWAS and the using of non-brain eQTL data may be the major reasons for the identification of only one significant gene in our study. Further investigations with larger sample size and using of brain eQTL data (of EAS) will help to validate this result and to identify more risk genes. Third, though our integrative analyses suggested that genetic variants may confer schizophrenia risk by regulating *TMEM180* expression, the functional risk variants (or causal variants) and how these functional variants regulate *TMEM180* expression remain unknown. Finally, despite our study revealed that *TMEM180* may have a role in neurodevelopment, currently we still do not know the exact role of *TMEM180* in brain development and schizophrenia. Further in vivo functional studies are needed to demonstrate how *TMEM180* confer risk of schizophrenia.

In summary, we performed a schizophrenia integrative analysis using genetic associations and eQTL data from EAS. Our study identified *TMEM180* as a novel schizophrenia risk gene whose expression alternation may have a role in schizophrenia. Further functional study will elucidate the role and mechanisms of *TMEM180* in schizophrenia.

## Supplementary Material

sbab032_suppl_Supplementary_Material
