# Supplementary material for: Integrative Analyses Followed by Functional Characterization Reveal TMEM180 as a Schizophrenia Risk Gene
Source: Schizophr Bull. 2021 Mar 26;47(5):1364–74. doi: 10.1093/schbul/sbab032 (PMC8379544; doi:10.1093/schbul/sbab032)
Supplement: sbab032_suppl_Supplementary_Material [file sbab032_suppl_supplementary_material.docx]

**Supplementary material for**

**Integrative analyses followed by functional characterization reveal *TMEM180* as a schizophrenia risk gene**

Jun-Yang Wang^1,2,6^, Xiao-Yan Li^1,2,6^, Hui-Juan Li^1,2,6^, Jie-Wei Liu^1^, Yong-Gang Yao^1-3,5^, Ming Li^1-3,5^, Xiao Xiao^1,7,*^, & Xiong-Jian Luo^1-4,7,*^

**Supplementary methods**

***Expression quantitative trait loci (eQTL) data of EAS***

In the original study, Stranger *et al*. quantified gene expression levels in lymphoblastoid cell lines of 726 individuals from 8 HapMap global populations using the Illumina’s whole genome expression array[^1^](#_ENREF_1). Of these subjects, 162 donors were EAS (80 Han Chinese from Beijing (CHB) and 82 Japanese in Tokyo (JPT)), and we downloaded the respective data from ArrayExpress: <https://www.ebi.ac.uk/arrayexpress/experiments/E-MTAB-264/>. Their corresponding genotype data (obtained with the Illumina Sentrix Human-6 Expression BeadChip) were downloaded from HapMap: <https://www.broadinstitute.org/medical-and-population-genetics/hapmap-3>. More details of RNA extraction, gene expression quantification, genotyping, quality control, and statistical analyses can be found in the original study[^1^](#_ENREF_1).

***Transcriptome-wide Association Study (TWAS)***

In this study, we firstly calculated SNP-expression weights using eQTL data of EAS (from the HapMap^[2](#_ENREF_2)^ or weblink). For a given gene, the associations between this gene and the SNPs surrounding it (within 1-Mb) were computed with FUSION.compute_weights.R script based on three predictive models included in FUSION (i.e., LASSO, top1, and Elastic Net)[^3^](#_ENREF_3). We used a strict Bonferroni-corrected *P* threshold to correct TWAS significant genes (i.e., TWAS *P* value = 6.84 × 10^−5^ (0.05/731)). The principles and detailed procedures of FUSION were provided in the original paper[^3^](#_ENREF_3).

***Summary-data based Mendelian Randomization (SMR) analysis***

Only SNP-gene associations with *P* values < 1 × 10^-5^ (--peqtl-smr 1e-5) were included in the SMR analysis. In addition, HEIDI test was also performed to test the presence of heterogeneity in the SMR association statistics[^4^](#_ENREF_4) and only genes passed HEIDI test (*P*_HEIDI_ ≥ 0.05) were retained. The significant associations were determined by a Bonferroni-adjusted significance level of 1.34 × 10^-4^ (i.e., 0.05/372) to account for multiple comparisons.

***Functional annotation of rs2902544***

We explored the functionality of rs2902544 using functional annotation tools RegulomeDB^[5](#_ENREF_5)^ and Alibaba2[^6^](#_ENREF_6). Briefly, RegulomeDB classifies SNPs into six categories based on their potential functional consequences (including their associations with gene expression (eQTL), location in binding motifs of transcription factors, disruption binding of transcription factors, Epigenomic annotation and etc). Low ranking indicates higher probability that the SNP is functional. For example, if a SNP has a RegulomeDB score of 1, suggesting this SNP is likely functional[^5^](#_ENREF_5). Accordingly, this SNP is more likely to be functional than other SNPs that have RegulomeDB scores > 1. In other words, SNPs with lower RegulomeDB scores were more likely to be functional variants. Alibabi2[^6^](#_ENREF_6) used TRANSFAC 3.5 public data to predict transcription factor binding of sequences[^6^](#_ENREF_6).

***Expression analysis of TMEM180 in peripheral blood of schizophrenia cases and controls (EAS sample)***

Briefly, 18 early-onset schizophrenia (EOS) patients and 12 healthy controls (all of the included subjects were Han Chinese) were included in the study by Sun *et al*.[^7^](#_ENREF_7). Whole-genome gene expression profiles in peripheral blood mononuclear cells were measured by using the Agilent Array platform and a total of 17 200 valid probes were used to quantify gene expression levels. We downloaded the gene expression data (GSE54913)[^7^](#_ENREF_7) generated by Sun *et al.* and performed differential gene expression using R software (Limma package). Student’s *t* test was used to compare if *TMEM180* mRNA expression level was statistically different in schizophrenia cases and controls. And the effect size of (Cohen's d, which equals to (M1-M2)/(Std dev) (Cohen, J. 1998. Statistical Power Analysis for the Behavioral Sciences. 2nd ed. Hillsdale, NJ: Lawrence Erlbaum Associates.). M1 and M2 represent the mean value of the group 1 and 2, respectively. Std dev represents the pooled standard deviation of the M1 and M2 groups) of differential expression results was calculated using rstatix R package[^8^](#_ENREF_8) based on the expression of *TMEM180* in cases and controls.

***Expression analysis of TMEM180 in brain tissues of schizophrenia cases and controls (European sample)***

Briefly, gene expression profiling in brain tissues of 559 schizophrenia cases and 936 controls were measured by RNA sequencing (RNA-Seq). Most of subjects were Caucasians (~70%) and African Americans (~22%), with 64.9% were males and 35.1% were females. The average age of cases and controls were 57 and 56 years, respectively.

***Spatio-temporal expression pattern of TMEM180 in developing and adult human brains***

To explore the potential role of *TMEM180* in the central nervous system, we explored the spatio-temporal expression pattern of *TMEM180* in developing and adult human brain using expression dataset from the BrainSpan (http://www.brainspan.org/)[^9^](#_ENREF_9). Gene expression values (based on RNA sequencing) of *TMEM180* in the prefrontal cortex (PFC) (N = 42) were downloaded and transformed as previously described[^10^](#_ENREF_10). The transformed expression level was used to plot the temporal expression pattern of *TMEM180* in developing and adult human brain.

***Knockdown assays***

shRNAs were cloned into pLKO.1-EGFP-Puro vector, which were then packaged into lentivirus in HEK-293T cells. Lentiviral particles were concentrated using Lenti-X Concentrator (Takala, Cat. No: 631231). Fourty-eight hours post infection, the cells were selected with 2 µg/mL puromycin (Sigma, Cat. No: 540222) for 14 days. Knockdown efficiency was determined by qPCR.

***Isolation and culture of mouse neural stem cells (mNSCs)***

The obtained brain tissues from embryonic day 13.5 mice were washed several times with ice-cold PBS containing 1% penicillin/streptavidin (100 units/mL of penicillin and 100 µg/mL of streptomycin) (Gibco, 15140-122). The washed tissues were then transferred into proliferation medium and dissociated with repeated pipetting. The dissociated cells were then filtered with a 400 Mesh strainer and transferred into a new 12-well plate (3 × 10^5^ cells/mL). After culturing for 3 to 5 days in proliferation medium, the generated neurospheres were characterized for further experiments. The proliferation medium consists of DMEM/F12 (1:1, Gibco, Cat.No: C11330500BT), 2% B27 supplement (Gibco, Cat.No: 12587010), 1% N2 supplement (Gibco, Cat.No: 17502048), 20 ng/mL pidermal growth factor (EGF, STEMCELL, Cat.No: 78006), 10 ng/mL basic fibroblast growth factor (bFGF, STEMCELL, Cat.No: 78003), 1% penicillin/streptavidin (100 units/mL of penicillin and 100 µg/mL of streptomycin) (Gibco, Cat.No: 15140-122), and 0.0002% heparin (20 µg/mL) (STEMCELL, Cat.No: 07980), as previously described[^11^](#_ENREF_11)^,^ [^12^](#_ENREF_12).

***EdU proliferation assay***

The mNSCs cells from the neurospheres were plated onto the 24-well plates (pre-coated with laminin (Sigma, Cat.No: L2020-1mg)) at a density of 3 × 10^5^ cells/mL. After culturing for 48 hours, 20 ug/mL EdU (5-Ethynyl-2'-deoxyuridine, RiboBio, Cat.No: C00053) solution was added (diluted by using proliferation medium) and incubated for 1 hour in cell culture incubator. The Cell-Light EdU Apollo567 In Vitro Kit (RiboBio, Cat.No: [C10310-1](https://www.ribobio.com/product_detail/?sku=C10310-1)) was used to detect the incorporation of EdU. Briefly, the mNSCs were firstly washed once with PBS and fixed with 4% Paraformaldehyde (PFA) for 15 min at room temperature. Then the cells were incubated with 2 mg/mL glycine for 5 min and permeabilized with PBST (1 × PBS containing 3% Triton X-100) for 15 min at room temperature. Each well was treated with Apollo®567 staining solution for 30 min in the dark and the EdU signal was detected with confocal microscope (Olympus, Japan, Cat.No: FV1000). DAPI (4′, 6-diamidino-2-phenylindole) (Beyotime, Cat.No: C1006) was used to label the DNA.

***Cell Counting Kit-8 (CCK-8) proliferation assays.***

The mNSCs cells were plated onto the 96-well plates (1.8 × 10^4^ cells/well, pre-coated with laminin (Sigma, Cat.No: L2020-1mg)). After culturing for 0-3 days (0, 24, 48 and 72 hours), CCK-8 (Beyotime, Cat.No: C0042) solution (10 μL/well) was added to each well and incubated for 2 hours. The absorbance value of each well at wavelength of 450 nm was measured with Spectrophotometer (BioTek, USA).

***Differentiation of mNSCs into neurons and astrocyte cells***

Differentiation medium was composed of following recipes: DMEM/F12 (Gibco, Cat.No: C11330500BT) medium containing 2% B27 supplement (Gibco, Cat.No: 12587010), 1% N2 supplement (Gibco, Cat.No: 17502048), 1% penicillin/streptavidin (100 units/mL of penicillin and 100 µg/mL of streptomycin) (Gibco, Cat.No: 15140-122), and 0.0002% heparin (20 µg/mL) (STEMCELL, Cat.No: 07980). After 3 days of spontaneous differentiation[^10^](#_ENREF_10)^,^ [^13^](#_ENREF_13), cells were fixed and immunofluorescence staining was performed to count MAP2 positive neurons and GFAP positive astrocytes.

***Immunofluorescence staining***

The mNSCs cells were fixed with 4% PFA for 15 min and washed three times with PBS at room temperature. Then the cells were permeabilized with PBST (1 × PBS containing 3% Triton X-100) for 15 min and blocked with blocking buffer (Beyotime, Cat.No: P0102) for 1 h at room temperature. Subsequently, the cells were incubated with primary antibodies overnight at 4℃, and incubated with secondary antibodies for 30 min (in the dark room) at room temperature. Before taking images, the cells were incubated with DAPI for 15 min to label DNA.

***Detailed information about the selected genes for qPCR validation***

*NPTX1* is highly expressed in the central nervous system, especially in the cerebellum, hippocampus, and cerebral cortex[^14^](#_ENREF_14)^,^ [^15^](#_ENREF_15) and previous study showed it plays an important role in excitatory synaptogenesis[^16^](#_ENREF_16). Intriguingly, previous studies showed that *NPTX1* was associated with Alzheimer’s disease and bipolar disorder[^17^](#_ENREF_17)^,^ [^18^](#_ENREF_18). *YWHAH* encodes one of the 14-3-3 family of proteins, which have been recognized as key regulators in cortical development[^19^](#_ENREF_19). In addition, *YWHAH* has been repeatedly reported to be associated with both schizophrenia and bipolar disorder[^20-22^](#_ENREF_20), indicating the pivotal role of this gene in neurodevelopment and psychiatric disorders. *GABRA2* **encodes** gamma-aminobutyric acid type A receptor alpha2 subunit, a gene previously reported to be associated with schizophrenia[^23^](#_ENREF_23). Intriguingly, a recent TWAS also showed that *GABRA2* was a schizophrenia risk gene[^24^](#_ENREF_24)*. COL26A1* mutation was previously reported to be associated with schizophrenia[^25^](#_ENREF_25). Finally, the protein product of *SLC6A9* is a glycine transporter, which regulates glycine levels and affects NMDAR-mediated neurotransmission (Cloning of the human glycine transporter type 1: molecular and pharmacological characterization of novel isoform variants and chromosomal localization of the gene in the human and mouse genomes). Interestingly, this gene is currently considered as a potential target for the treatment of negative symptoms of schizophrenia[^26-28^](#_ENREF_26).

***Antibodies used in this study***

Primary antibodies were: PAX6 (Milipore, 1:500, ab2237), NESTIN (Chemicon, 1:500, mab353), SOX2 (Santa cruze, 1:200, sc-17320), GFAP (Sigma, 1:1000, G9269) and MAP2 (Millipore, 1:200, AB5622). The secondary antibodies were: Alexa Fluor 488 donkey anti-rabbit (Life technology, 1:500, A32790), Alexa Fluor 555 donkey anti-mouse (Life technology, 1:500, A31570), Alexa Fluor 555 donkey anti-rabbit (Life technology, 1:500, A31572) and Alexa Fluor 647 donkey anti-goat (Life technology, 1:500, A32849).

***Transcriptome analysis***

Total RNA was isolated using TRIzol RNA Isolation Reagents (Life technologies, Cat.No: 15596018). RNA purity was assessed with the kaiaoK5500®Spectrophotometer (Kaiao, China). RNA integrity and concentration were evaluated using the RNA Nano 6000 Assay Kit of the Bioanalyzer 2100 system (Agilent technologies, USA). Sequencing libraries were prepared using the NEBNext® Ultra™ RNA Library Prep Kit for Illumina (NEB, Cat.No: E7530L). Libraries were sequenced to generate 150 bp paired-end reads with the Illumina NovaSeq 6000 platform. Differential expressed genes were identified using DESeq2 package[^29^](#_ENREF_29) implemented in Bioconductor based on fold change > 1.5 and adjusted *P* < 0.05. ClusterProfiler^[30](#_ENREF_30)^ were used to enrichment analyses (Kyoto Encyclopedia of Genes and Genomes (KEGG) and Gene Ontology (GO)).

***Real-time quantitative PCR***

cDNA was generated from 1 µg RNA with PrimeScript RT reagent Kit with gDNA Eraser (Takala, Cat.No: RR047B). We used 1 µL cDNA (1:5 dilution) as template for Real-time quantitative PCR (RT-qPCR). RT-qPCR was performed using TB Green® Premix Ex Taq™ II (Tli RNase H Plus) (Takala, Cat.No: RR820B) in 10 μL of reaction mixture. The *Actb* gene was used as the internal control. The relative changes in gene expression were analyzed using the 2^-ΔΔ^*^C^*^t^ method[^31^](#_ENREF_31).

**Supplemental Discussion**

**The potential role of TMEM180 in the brain:**

TMEM180 encodes a cation symporter that belongs to the glycoside–pentoside–hexuronide (GPH): cation symporter family[^32^](#_ENREF_32)^,^ [^33^](#_ENREF_33). To date, the exact cation transported by *TMEM180* remains unclear. Our GO analysis results showed that the differentially expressed genes affected by *TMEM180* knockdown were enriched in divalent metal ion transport, divalent inorganic cation transport, calcium ion transport into cytosol, synaptic transmission and other biological processes. Divalent cations play an important role in the CNS. For example, calcium flows into the cytoplasm through voltage-gated calcium channels can triggers vesicle release[^34-36^](#_ENREF_34). Thus, it is possible that *TMEM180* may regulate downstream genes through affecting the transport of divalent cations, which plays an important role in regulating gene expression and signaling transduction.

*TMEM180* is widely expressed in different human tissues, with relative higher expression in brain tissues (data from GTEx^[37](#_ENREF_37)^, **supplementary figure 3**). In addition, expression of *TMEM180* (data from the BrainSpan: http://www.brainspan.org/) was relatively higher at early developmental stage compared with childhood and adulthood stages (**supplementary figure 4**). Cell-type-specific expression analysis (detailed information were provided in **supplementary methods**) showed that *TMEM180* did not exhibit a cell-type-specific expression in mouse and human brains (**supplementary figure 5**). These expression data suggested that *TMEM180* may have a pivotal role in the central nervous system and the genes regulated by *TMEM180* are relatively conserved in mouse and human.

***Discussion on ancestry-specific findings diverge and converge across modalities in schizophrenia:***

Though GWAS and integrative analyses performed in European populations did not identify *TMEM180* as a schizophrenia risk gene, *TMEM180* expression was dysregulated in schizophrenia cases in the PsychENCODE (the brain tissues were mainly from Europeans). Besides, functional explorations revealed the important role of *TMEM180* in neurodevelopment, a vital process that was recognized to be dysregulated in schizophrenia. Moreover, linkage disequilibrium analysis showed that the LD pattern of this genomic region surrounding rs2902544 is similar between CEU and CHB populations (**supplementary figure 8** and **9**). Finally, we noticed that 16 out of the top 30 differentially expressed genes (affected by *TMEM180* knockdown) showed expression change in schizophrenia cases compared with controls in the PsychENCODE (uncorrected *P* < 0.05) (**supplementary table 5**), implicating the convergence of the modalities.

**Supplementary figures 1-9:**

**
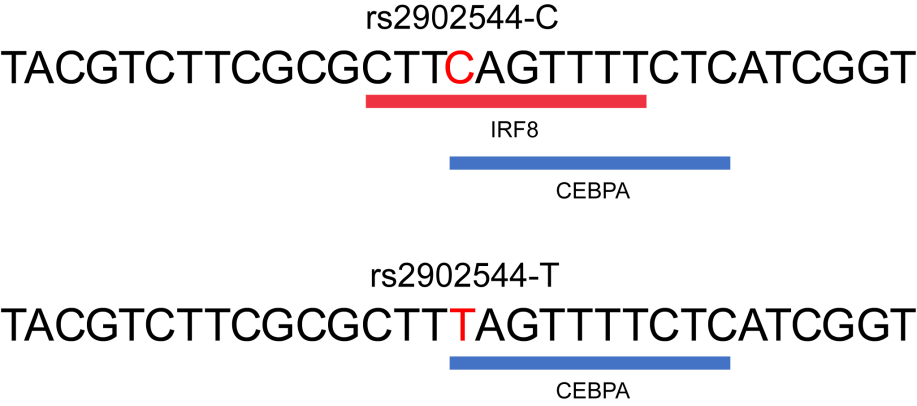
**

**Supplementary figure 1. Different alleles of rs2902544 may affect transcription factors binding.** Binding to transcription factors was predicted by the Alibaba2 (http://gene-regulation.com/pub/programs/alibaba2/index.html)[^6^](#_ENREF_6). The C allele binds to IRF8, while the T allele binds to CEBPA.


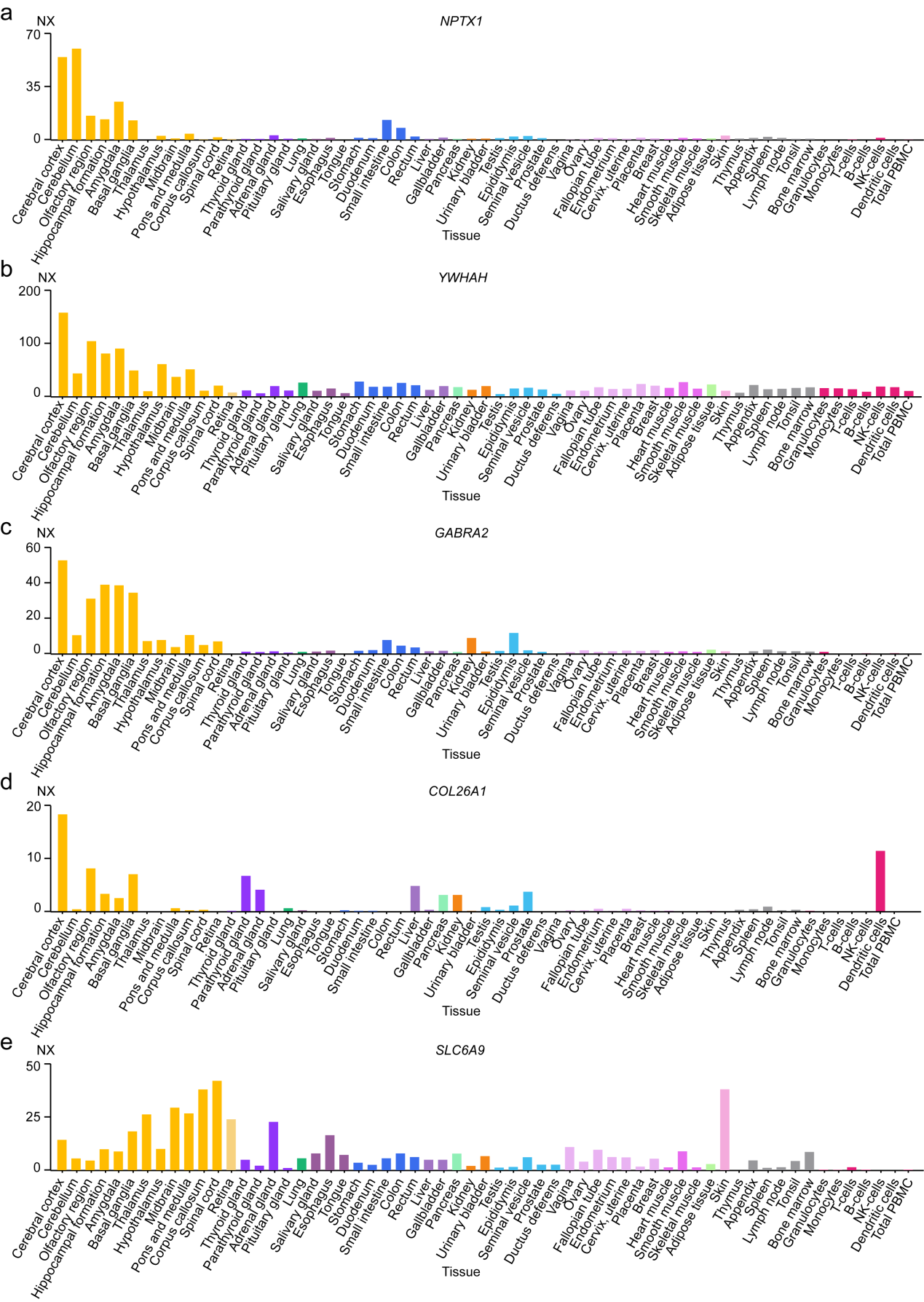


**Supplementary figure 2. The tissue-specific expression pattern of five genes for qPCR validation.** These 5 genes were from the top 30 differentially expressed genes of RNA-seq. **(a-e)** *NPTX1*, *YWHAH*, *GABRA2, COL26A1* and *SLC6A9* are abundantly expressed in the human brain. The tissue-specific expression data are from a public database (https://www.proteinatlas.org/) that combined the data from three transcriptome datasets: Human Protein Atlas (HPA), Genotype-Tissue Expression (GTEx) and Functional Annotation Of The Mammalian Genome 5 (FANTOM5). The y axis indicates normalized expression (NX) levels. The x axis shows 55 tissue types and 6 blood cell types. One color indicates one tissue group.


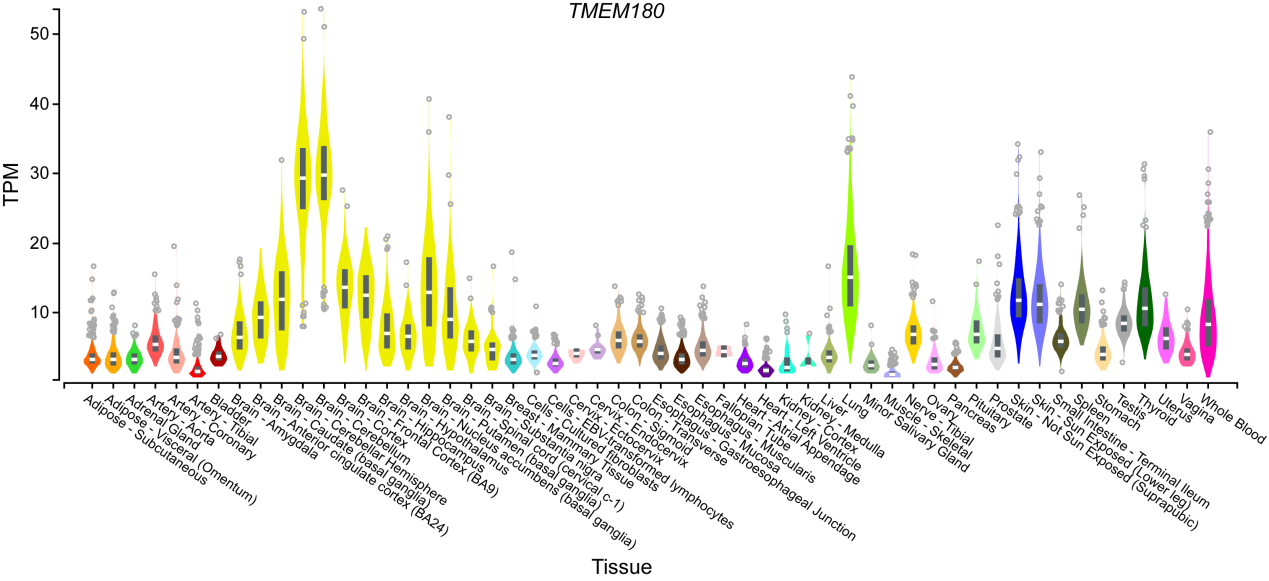


**Supplementary figure 3. The tissue-specific expression pattern of *TMEM180* in human tissues.** The tissue-specific expression data are from GTEx^[37](#_ENREF_37)^ (https://gtexportal.org/). The x axis shows 54 tissue types. Each color corresponds to a tissue group.


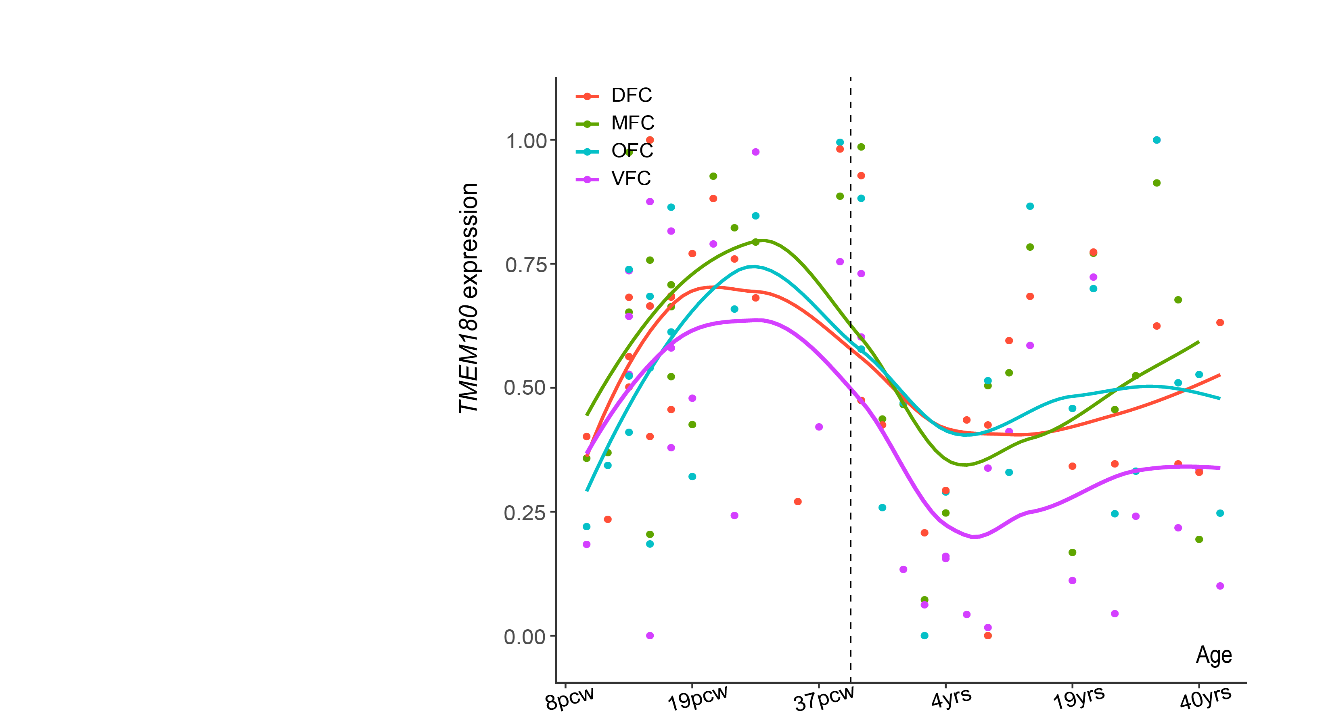


**Supplementary figure 4. Temporal expression patterns of *TMEM180* in human frontal cortex.** Expression of *TMEM180* was relatively higher at early developmental stage compared with childhood and adulthood stages (data from the BrainSpan).


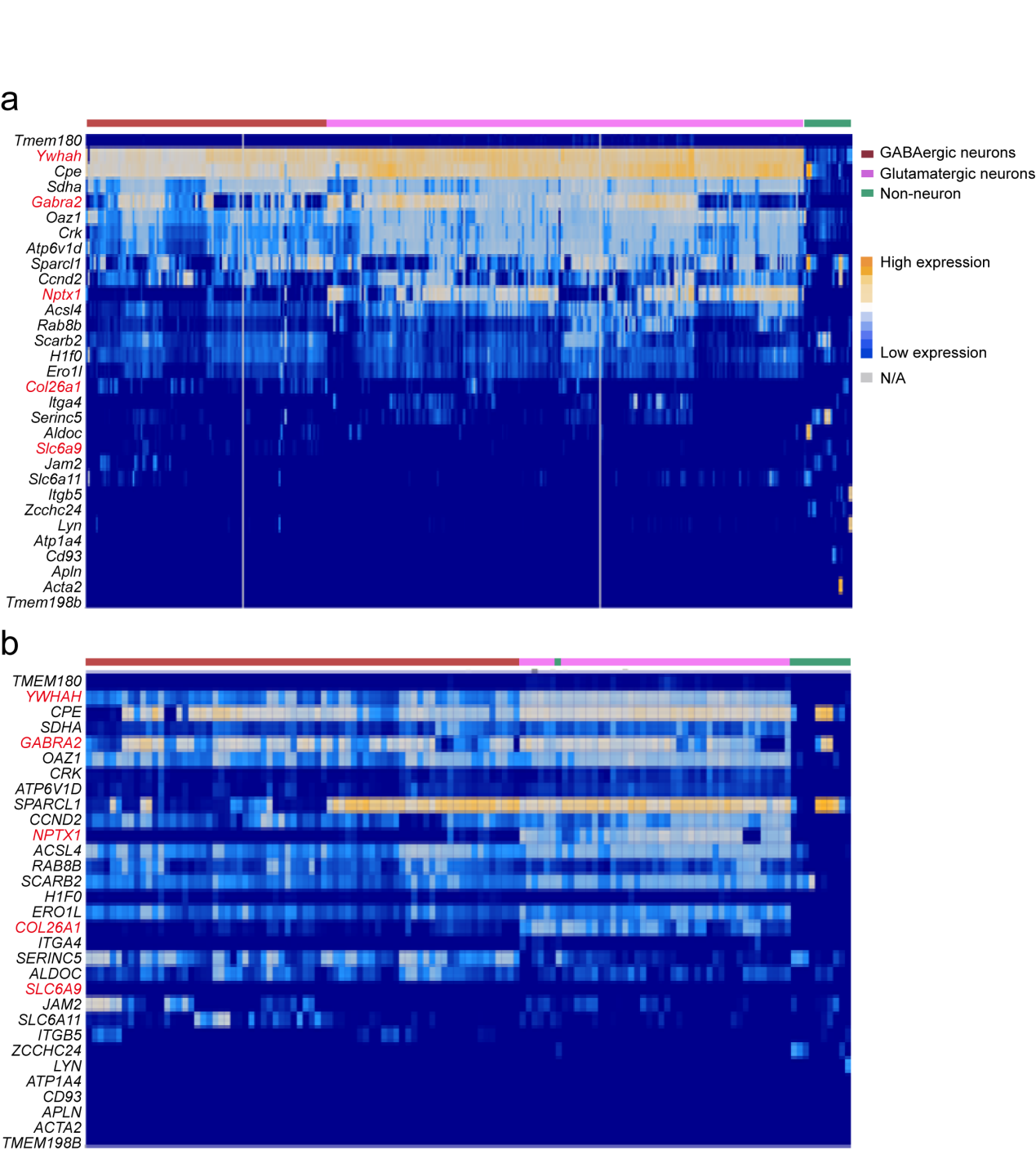


**Supplementary figure 5. The brain-cell-specific expression pattern of *TMEM180* and the top 30 differentially expressed genes (affected by *TMEM180* knockdown, based on RNA-seq).** The brain-cell-specific expression data were accessed through the Allen Brain Atlas for human **(a)** (https://celltypes.brain-map.org/rnaseq/human_m1_10x) and mouse brains **(b)** (<https://celltypes.brain-map.org/rnaseq/mouse_ctx-hip_10x>). Genes, marked by red color, were selected for qPCR verification.


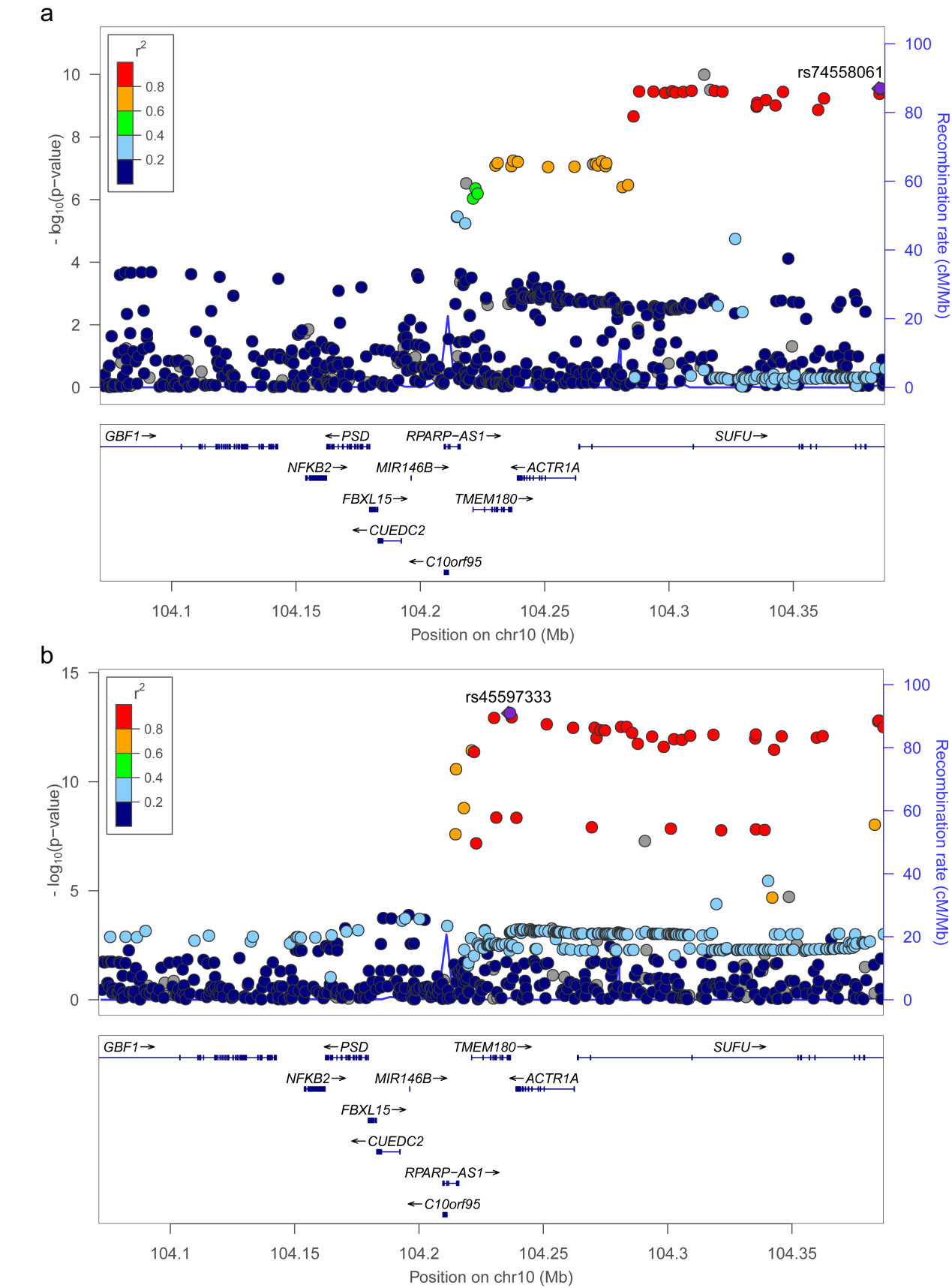


**Supplementary figure 6. The association between *TMEM180* and schizophrenia in EAS and EUR. (a)** The locus zoom plot showing the associations between variants in genomic region (300 kb) containing *TMEM180* and SCZ in European sample[^38^](#_ENREF_38). **(b)** The locus zoom plot showing the associations between variants in genomic region (300 kb) containing *TMEM180* and SCZ in EAS sample[^39^](#_ENREF_39).


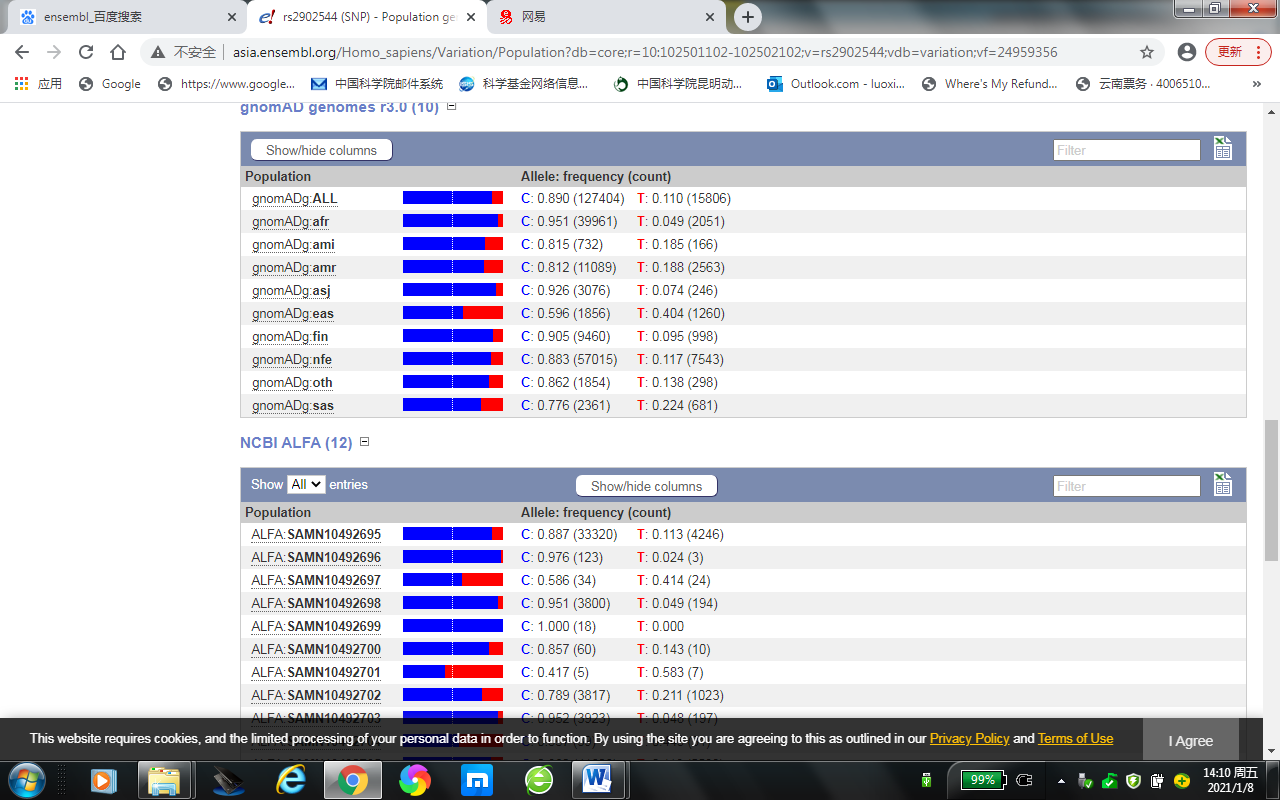


**Supplementary figure 7. The frequency of the C allele (risk allele) of rs2902544 in world populations.** Data were from the Ensembl website ([www.ensembl.org/](http://www.baidu.com/link?url=OEkNvsJg4Lz0HsZY3IT9zdiuqlOQxN3w09ct0Ml5lWRoapXk92QD7LBaZj-DlkQ7)). ALL, all genomeAD genomes individuals; afr, Africans; ami, Amish; amr, Latino/admixed American; asj, Ashkeniza Jewish; eas, East Asian; fin, Finnish; nfe, non-Finnish European; oth, other; sas, South Asian.


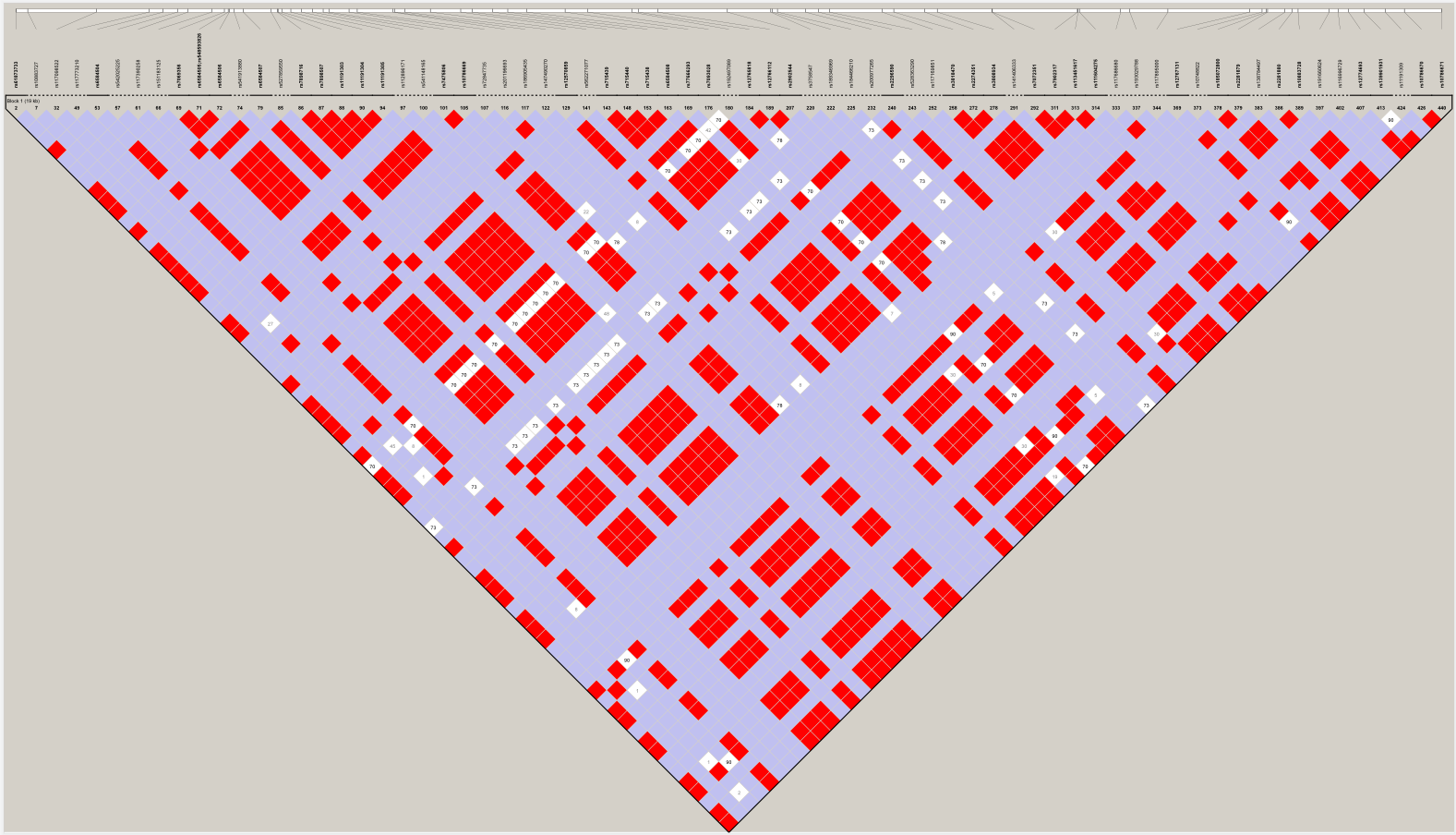


**Supplementary figure 8. Linkage disequilibrium heatmap of SNPs around rs2902544 (± 10 kb) in 1000 genome CEU population**


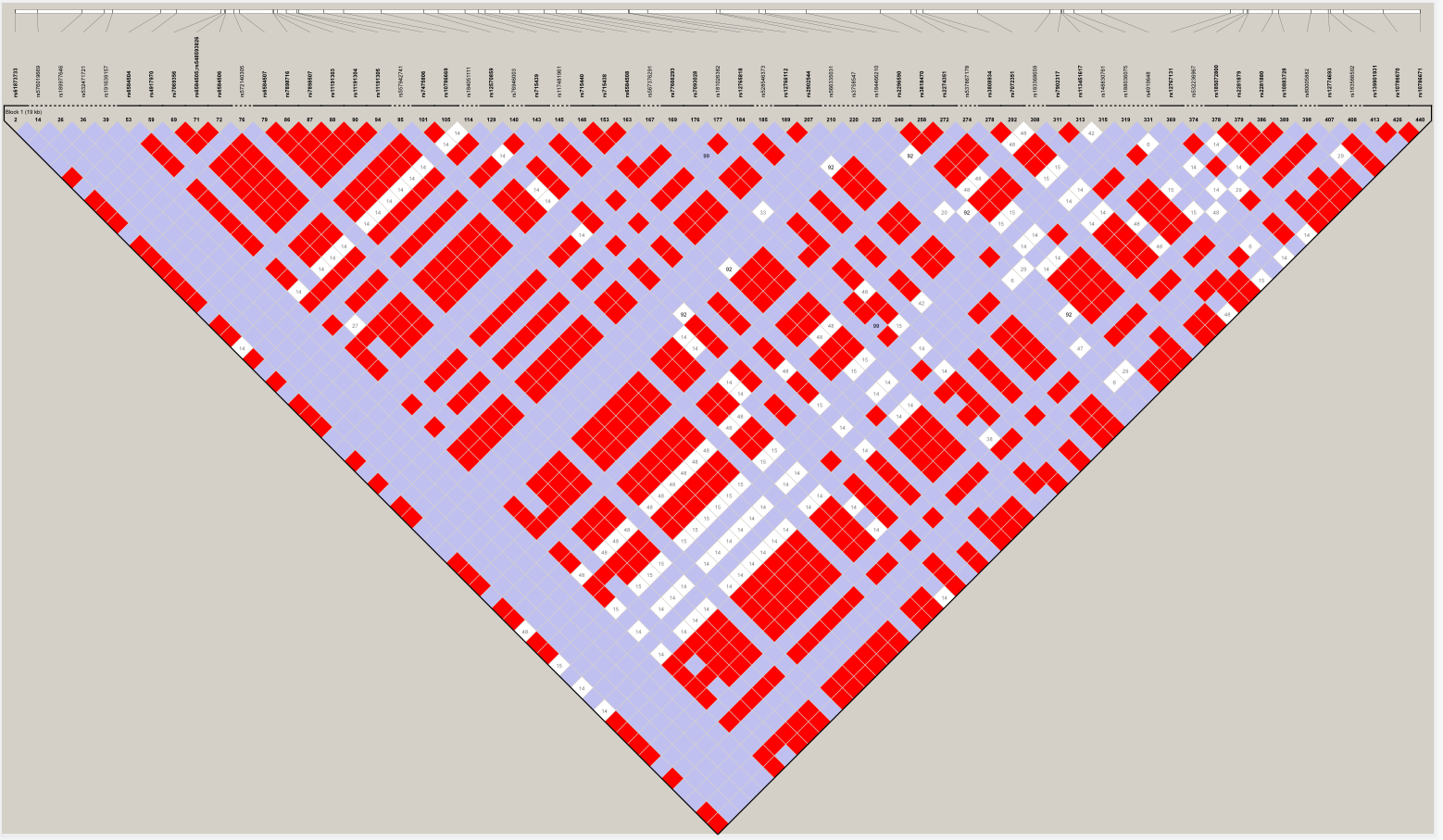


**Supplementary figure 9. Linkage disequilibrium heatmap of SNPs around rs2902544 (± 10 kb) in 1000 genome CHB population**

**Supplementary tables 1-5:**

**Supplementary table 1. Primers and shRNA sequences** **used in this study**

| Primers and shRNA | Sequence |
| --- | --- |
| *Tmem180*-shRNA#1-F | CCGGGCTGGCTTGCAATTCTTACTGCTCGAGCAGTAAGAA  TTGCAAGCCAGCTTTTTG |
| *Tmem180*-shRNA#1-R | AATTCAAAAAGCTGGCTTGCAATTCTTACTGCTCGAGCAG  TAAGAATTGCAAGCCAGC |
| *Tmem180*-shRNA#2-F | CCGGGGTACTGAACCATCGGAAGCACTCGAGTGCTTCCGA  TGGTTCAGTACCTTTTTG |
| *Tmem180*-shRNA#2-R | AATTCAAAAAGGTACTGAACCATCGGAAGCACTCGAGTGC  TTCCGATGGTTCAGTACC |
| *Tmem180*-qPCR-F | GTCACTTCAACAGTAACTTCTTCCC |
| *Tmem180*-qPCR-R | ACATAGGAGATGCCCAACAGG |
| *Actb*-qPCR-F | GGCTGTATTCCCCTCCATCG |
| *Actb*-qPCR-R | CCAGTTGGTAACAATGCCATGT |
| *Nptx1*-qPCR-F | GTGCTGGGTCAGGAACA |
| *Nptx1*-qPCR-R | CAGATGTTGAAATGGGCTA |
| *Ywhah*-qPCR-F | TTGGAGGGTTATTAGTAGCA |
| *Ywhah*-qPCR-R | AGCAGAGCCAAGACATCA |
| *Gabra2*-qPCR-F | AGCACATGCAATGTATGGTCTC |
| *Gabra2*-qPCR-R | GATGGTGATGTTATTTTTAGCCT |
| *Col26a1*-qPCR-F | AACCTCGTGAGGACTCTCAT |
| *Col26a1*-qPCR-R | CGTTCACTCATGTCGCTAA |
| *Slc6a9*-qPCR-F | AAAAGGTGCCAAAGGGAT |
| *Slc6a9*-qPCR-R | GCAGAGGTATGGGAAACG |
| *Gfap*-qPCR-F | CGGAGACGCATCACCTCTG |
| *Gfap*-qPCR-R | AGGGAGTGGAGGAGTCATTCG |
| *Map2*-qPCR-F | GCCAGCCTCAGAACAAACA |
| *Map2*-qPCR-R | GCTCAGCGAATGAGGAAGGA |

**Supplementary table 3. Association significance between *TMEM180* and schizophrenia in a recent TWAS of European populations**

| **Gene** | **CHR** | **Best.GWAS.ID^a^** | **A1/A2** | **OR^b^** | **eQTL ID^c^** | **TWAS.Z^d^** | **TWAS.P** |
| --- | --- | --- | --- | --- | --- | --- | --- |
| *TMEM180* | 10 | rs7475853 | A/G | 0.87 | rs7094906 | -2.751 | 5.95E-03 |

^a^The SNP that showed the most significant association with schizophrenia in this locus. ^b^Odds ratio is based on A1. ^c^The SNP that showed the most significant association with gene expression in this locus. ^d^The *Z* statistic reflects the association strength between this gene and schizophrenia. *Z* < 0 suggests that this gene was predicted to be down-regulated in schizophrenia cases compared with controls, and vice versa. Data are from a previous study[^40^](#_ENREF_40).

**Supplementary table 4. Association between rs2902544 and schizophrenia in EAS and EUR populations**

| **SNP** | **Chr** | **A_1_** | **A_2_** | **OR^a^** | **SE^b^** | **Beta** | **P** | **study** |
| --- | --- | --- | --- | --- | --- | --- | --- | --- |
| rs2902544 | chr10:104261359 | T | C | 0.92 | 0.015 | -0.08 | 9.12E-08 | PGC2+Clozuk[^38^](#_ENREF_38) |
|  |  |  |  | 0.91 | 0.014 | -0.10 | 3.45E-13 | Lam et al[^39^](#_ENREF_39) |

^a^Odds ratio is based on A_1_. ^b^Standard error of the ln(OR). Data are from previous studies[^38^](#_ENREF_38)^,^ [^39^](#_ENREF_39).

**Supplementary table 5. Differential expression analysis of the top 30 genes affected by *Tmem180* knockdown in the PsychENCODE dataset**

| **Gene symbol** | **Chr** | **Start** | **Stop** | ***P* value** | **FDR^a^** |
| --- | --- | --- | --- | --- | --- |
| *SCARB2* | chr4 | 77079890 | 77135046 | 2.38E-05 | 5.24E-04 |
| *CPE* | chr4 | 166282346 | 166419472 | 2.24E-04 | 3.00E-03 |
| *GABRA2* | chr4 | 46250444 | 46477247 | 9.02E-04 | 8.62E-03 |
| *SDHA* | chr5 | 218356 | 256815 | 1.62E-03 | 1.34E-02 |
| *SLC6A11* | chr3 | 10857885 | 10982419 | 2.04E-03 | 1.60E-02 |
| *JAM2* | chr21 | 27011584 | 27089874 | 4.20E-03 | 2.77E-02 |
| *ERO1L* | chr14 | 53106634 | 53162618 | 5.30E-03 | 3.29E-02 |
| *ITGA4* | chr2 | 182321929 | 182400914 | 2.06E-02 | 8.86E-02 |
| *ALDOC* | chr17 | 26900133 | 26904282 | 2.48E-02 | 1.01E-01 |
| *SPARCL1* | chr4 | 88394487 | 88452213 | 2.71E-02 | 1.07E-01 |
| *APLN* | chrX | 128779240 | 128788933 | 3.14E-02 | 1.19E-01 |
| *NPTX1* | chr17 | 78440948 | 78451643 | 3.58E-02 | 1.30E-01 |
| *SERINC5* | chr5 | 79407050 | 79551898 | 3.75E-02 | 1.35E-01 |
| *SLC6A9* | chr1 | 44457172 | 44497139 | 4.00E-02 | 1.41E-01 |
| *RAB8B* | chr15 | 63481668 | 63559981 | 4.17E-02 | 1.45E-01 |
| *CD93* | chr20 | 23059986 | 23066977 | 4.97E-02 | 1.64E-01 |
| *ZCCHC24* | chr10 | 81142081 | 81205383 | 5.44E-02 | 1.74E-01 |
| *CRK* | chr17 | 1323983 | 1366456 | 9.07E-02 | 2.47E-01 |
| *ACSL4* | chrX | 108867473 | 108976632 | 9.62E-02 | 2.56E-01 |
| *ATP1A4* | chr1 | 160121360 | 160156767 | 1.09E-01 | 2.79E-01 |
| *TMEM198B* | chr12 | 56223529 | 56230030 | 1.15E-01 | 2.89E-01 |
| *ACTA2* | chr10 | 90694831 | 90751147 | 1.16E-01 | 2.91E-01 |
| *ATP6V1D* | chr14 | 67761088 | 67826982 | 2.64E-01 | 4.88E-01 |
| *LYN* | chr8 | 56792372 | 56923940 | 2.76E-01 | 5.02E-01 |
| *OAZ1* | chr19 | 2269485 | 2273487 | 2.80E-01 | 5.06E-01 |
| *YWHAH* | chr22 | 32340447 | 32353590 | 3.41E-01 | 5.71E-01 |
| *COL26A1* | chr7 | 101006101 | 101202304 | 3.78E-01 | 6.06E-01 |
| *ITGB5* | chr3 | 124480795 | 124620265 | 5.78E-01 | 7.64E-01 |
| *H1F0* | chr22 | 38201114 | 38203442 | 6.70E-01 | 8.27E-01 |
| *CCND2* | chr12 | 4382938 | 4414516 | 7.73E-01 | 8.88E-01 |

^a^FDR-corrected q value. Data are from PsychENCODE dataset (<http://resource.psychencode.org/>).

**References**

1. Stranger BE, Montgomery SB, Dimas AS, et al. Patterns of Cis Regulatory Variation in Diverse Human Populations. *Plos Genet.* 2012;8(4):272-284.

2. Altshuler D, Brooks LD, Chakravarti A, et al. A haplotype map of the human genome. *Nature.* 2005;437(7063):1299-1320.

3. Gusev A, Ko A, Shi H, et al. Integrative approaches for large-scale transcriptome-wide association studies. *Nat Genet.* 2016;48(3):245-252.

4. Zhu ZH, Zhang FT, Hu H, et al. Integration of summary data from GWAS and eQTL studies predicts complex trait gene targets. *Nat Genet.* 2016;48(5):481-487.

5. Boyle AP, Hong EL, Hariharan M, et al. Annotation of functional variation in personal genomes using RegulomeDB. *Genome Res.* 2012;22(9):1790-1797.

6. Grabe N. AliBaba2: Context specific identification of transcription factor binding sites. *In Silico Biology.* 2002;2(1):S1-S15.

7. Sun L, Cheng Z, Zhang F, Xu Y. Gene expression profiling in peripheral blood mononuclear cells of early-onset schizophrenia. *Genomics data.* 2015;5:169-170.

8. Kassambara A. Rstatix: Pipe-friendly framework for basic statistical tests in R.*https://githubcom/kassambara/rstatix.* 2019b.

9. Kang HJ, Kawasawa YI, Cheng F, et al. Spatio-temporal transcriptome of the human brain. *Nature.* 2011;478(7370):483-489.

10. Yang CP, Li XY, Wu Y, et al. Comprehensive integrative analyses identify GLT8D1 and CSNK2B as schizophrenia risk genes. *Nat Commun.* 2018;9:838.

11. Louis SA, Mak CK, Reynolds BA. Methods to culture, differentiate, and characterize neural stem cells from the adult and embryonic mouse central nervous system. *Methods in molecular biology.* 2013;946:479-506.

12. Li JY, Liu J, Manaph NPA, Bobrovskaya L, Zhou XF. ProBDNF inhibits proliferation, migration and differentiation of mouse neural stem cells. *Brain research.* 2017;1668:46-55.

13. Kong H, Fan Y, Xie J, et al. AQP4 knockout impairs proliferation, migration and neuronal differentiation of adult neural stem cells. *J Cell Sci.* 2008;121(24):4029-4036.

14. Persson A, Hober S, Uhlen M. A human protein atlas based on antibody proteomics. *Curr Opin Mol Ther.* 2006;8(3):185-190.

15. Schlimgen AK, Helms JA, Vogel H, Perin MS. Neuronal Pentraxin, a Secreted Protein with Homology To Acute-Phase Proteins Of the Immune-System. *Neuron.* 1995;14(3):519-526.

16. Xu DS, Hopf C, Reddy R, et al. Narp and NP1 form heterocomplexes that function in developmental and activity-dependent synaptic plasticity. *Neuron.* 2003;39(3):513-528.

17. Cummings DM, Benway TA, Ho H, et al. Neuronal and Peripheral Pentraxins Modify Glutamate Release and may Interact in Blood-Brain Barrier Failure. *Cereb Cortex.* 2017;27(6):3437-3448.

18. Rajkumar AP, Christensen JH, Mattheisen M, et al. Analysis of t(9;17)(q33.2; q25.3) chromosomal breakpoint regions and genetic association reveals novel candidate genes for bipolar disorder. *Bipolar Disord.* 2015;17(2):205-211.

19. Cornell B, Toyo-Oka K. 14-3-3 Proteins in Brain Development: Neurogenesis, Neuronal Migration and Neuromorphogenesis. *Front Mol Neurosci.* 2017;10:318.

20. Duan SW, Gao R, Xing QH, et al. A family-based association study of schizophrenia with polymorphisms at three candidate genes. *Neurosci Lett.* 2005;379(1):32-36.

21. Oldmeadow C, Mossman D, Evans TJ, et al. Combined analysis of exon splicing and genome wide polymorphism data predict schizophrenia risk loci. *J Psychiatr Res.* 2014;52:44-49.

22. Grover D, Verma R, Goes FS, et al. Family-Based Association of YWHAH in Psychotic Bipolar Disorder. *Am J Med Genet B.* 2009;150B(7):977-983.

23. Petryshen TL, Middleton FA, Tahl AR, et al. Genetic investigation of chromosome 5q GABA(A) receptor subunit genes in schizophrenia. *Mol Psychiatr.* 2005;10(12):1074-1088.

24. Hall LS, Medway CW, Pain O, et al. A transcriptome-wide association study implicates specific pre- and post-synaptic abnormalities in schizophrenia. *Human molecular genetics.* 2020;29(1):159-167.

25. Li QQ, Wineinger NE, Fu DJ, et al. Genome-wide association study of paliperidone efficacy. *Pharmacogenet Genom.* 2017;27(1):7-18.

26. Javitt DC. Glycine transport inhibitors for the treatment of schizophrenia: Symptom and disease modification. *Curr Opin Drug Disc.* 2009;12(4):468-478.

27. Pinard E, Alanine A, Alberati D, et al. Selective GlyT1 Inhibitors: Discovery of [4-(3-Fluoro-5-trifluoromethylpyridin-2-yl)piperazin-1-yl]-[5-methanesulfonyl-2-((S)-2,2,2-trifluoro-1-methylethoxy)phenyl]methanone (RG1678), a Promising Novel Medicine To Treat Schizophrenia. *J Med Chem.* 2010;53(12):4603-4614.

28. Kingwell K. Schizophrenia drug gets negative results for negative symptoms. *Nat Rev Drug Discov.* 2014;13(4):244-245.

29. Love MI, Huber W, Anders S. Moderated estimation of fold change and dispersion for RNA-seq data with DESeq2. *Genome Biol.* 2014;15(12):550.

30. Yu GC, Wang LG, Han YY, He QY. clusterProfiler: an R Package for Comparing Biological Themes Among Gene Clusters. *Omics.* 2012;16(5):284-287.

31. Livak KJ, Schmittgen TD. Analysis of relative gene expression data using real-time quantitative PCR and the 2(T)(-Delta Delta C) method. *Methods.* 2001;25(4):402-408.

32. Saier MH, Reddy VS, Tsu BV, et al. The Transporter Classification Database (TCDB): recent advances. *Nucleic Acids Res.* 2016;44(D1):D372-D379.

33. Anzai T, Matsumura Y. Topological analysis of TMEM180, a newly identified membrane protein that is highly expressed in colorectal cancer cells. *Biochem Bioph Res Co.* 2019;520(3):566-572.

34. Stanley EF. Single Calcium Channels And Acetylcholine-Release at a Presynaptic Nerve-Terminal. *Neuron.* 1993;11(6):1007-1011.

35. Dunlap K, Luebke JI, Turner TJ. Exocytotic Ca2+ Channels In Mammalian Central Neurons. *Trends Neurosci.* 1995;18(2):89-98.

36. Olivera BM, Miljanich GP, Ramachandran J, Adams ME. Calcium-Channel Diversity And Neurotransmitter Release - the Omega-Conotoxins And Omega-Agatoxins. *Annu Rev Biochem.* 1994;63:823-867.

37. Ardlie KG, DeLuca DS, Segre AV, et al. The Genotype-Tissue Expression (GTEx) pilot analysis: Multitissue gene regulation in humans. *Science.* 2015;348(6235):648-660.

38. Pardinas AF, Holmans P, Pocklington AJ, et al. Common schizophrenia alleles are enriched in mutation-intolerant genes and in regions under strong background selection. *Nat Genet.* 2018;50(3):381-389.

39. Lam M, Chen CY, Li ZQ, et al. Comparative genetic architectures of schizophrenia in East Asian and European populations. *Nat Genet.* 2019;51(12):1670-1678.

40. Gandal MJ, Zhang P, Hadjimichael E, et al. Transcriptome-wide isoform-level dysregulation in ASD, schizophrenia, and bipolar disorder. *Science.* 2018;362(6420):eaat8127.
